# Cigarette Smoke Exposure Alters mSin3a and Mi-2α/β Expression; implications in the control of pro-inflammatory gene transcription and glucocorticoid function

**DOI:** 10.1186/1476-9255-7-33

**Published:** 2010-07-16

**Authors:** John A Marwick, Christopher S Stevenson, Kian Fan Chung, Ian M Adcock, Paul A Kirkham

**Affiliations:** 1 Section of Airways Disease, National Heart & Lung Institute, Imperial College London, UK; 2 Respiratory Disease Area, Novartis Institute for Biomedical Research, Horsham, UK; 3 Respiratory Pharmacology, National Heart & Lung Institute, Imperial College London, UK

## Abstract

**Background:**

The key co-repressor complex components HDAC-2, Mi-2α/β and mSin3a are all critical to the regulation of gene transcription. HDAC-2 function is impaired by oxidative stress in a PI3Kδ dependant manner which may be involved in the chronic glucocorticoid insensitive inflammation in the lungs of COPD patients. However, the impact of cigarette smoke exposure on the expression of mSin3a and Mi2α/β and their role in glucocorticoid responsiveness is unknown.

**Methods:**

Wild type, PI3Kγ knock-out (PI3Kγ^-/-^) and PI3K kinase dead knock-in (PI3Kδ^D910/A910^) transgenic mice were exposed to cigarette smoke for 3 days and the expression levels of the co-repressor complex components HDAC-2, mSin3a, Mi-2α and Mi-2β and HDAC-2 activity in the lungs were assessed.

**Results:**

Cigarette smoke exposure impaired glucocorticoid function and reduced HDAC-2 activity which was protected in the PI3Kδ^D910/A910 ^mice. Both mSin3a and Mi-2α protein expression was reduced in smoke-exposed mice. Budesonide alone protected mSin3a protein expression with no additional effect seen with abrogation of PI3Kγ/δ activity, however Mi-2α, but not Mi-2β, expression was protected in both PI3Kδ^D910/A910 ^and PI3Kγ^-/- ^budesonide-treated smoke-exposed mice. The restoration of glucocorticoid function coincided with the protection of both HDAC activity and mSin3a and Mi-2α protein expression.

**Conclusions:**

Cigarette smoke exposure induced glucocorticoid insensitivity and alters co-repressor activity and expression which is prevented by blockade of PI3K signaling with glucocorticoid treatment. Inhibition of PI3Kδ signalling in combination with glucocorticoid treatment may therefore provide a therapeutic strategy for restoring oxidant-induced glucocortiocid unresponsiveness.

## Introduction

Gene transcription is tightly regulated by a highly complex and dynamic set of processes central to which is the recruitment of co-repressors to promoter bound sequence specific transcription factors [1-3]. Two of the major co-repressor complexes in mammalian cells are the mammalian Sin3a (mSin3a) and Mi-2/nucleosome remodelling and deacetylase (NuRD) complex, both of which are ubiquitously expressed [2,4-6]. The mammalian genome encodes two Mi-2 proteins; Mi-2α (encoded by the Chd3 gene) and Mi-2β (encoded by the Chd4 gene). Although the latter is predominantly associated with the NuRD complex they are structurally similar and no functional or cell type specific differentiation between Mi-2α and Mi-2β has yet been made [6].

Both the mSin3a and Mi-2/NuRD co-repressor complexes are large multi-component complexes in which not all of the components and their functions have been identified [2,6]. However, two of the key components include histone deacetylases 1 and 2 (HDAC1/2) and methyl transferases (including methyl-CpG-binding proteins) [2,7]. These are used to manipulate the basal transcriptional machinery and the chromatin structure through altering their acetylation and methylation status and thereby regulating gene expression [8]. In addition, Mi-2 possesses an ATPase-dependant nucleosome remodelling capacity and mSin3a can recruit sequence specific repressive transcription factors such as *Krüppel*-like transcription factor (KLF) 11 [4,9].

HDACs are central in the regulation of pro-inflammatory gene transcription mediated by nuclear hormone receptors including the glucocorticoid receptor α (GRα) [10-15]. HDACs function by deacetylating of key components of the transcriptional machinery including the core histone proteins resulting in their in re-association with the DNA, thus presenting a transcriptionally closed conformation [1,16].

HDAC-2 function is impaired by oxidative stress which may be critical in the development of the uncontrolled chronic and relatively glucocorticoid insensitive inflammation seen in the lungs of patients with chronic obstructive pulmonary disease (COPD) [11,17-19].

The impact of oxidative stress on key components of the co-repressor complexes have only just started to be explored, with the very recent publication highlighting the impact of oxidative stress driven protein kinase-CK2 activation on co-repressor activity and HDAC2 function [20]. Nevertheless, the impact is still largely unknown but may be important for the development of both uncontrolled inflammatory responses and the impairment of glucocorticoid function. In addition, we previously demonstrated that abolition of PI3K signalling restores both HDAC activity and glucocorticoid responsiveness in smoke exposed mice [21]. The impact of PI3K signalling on other components of GR-associated co-repressor complexes is also unknown.

In this study we look at the impact of cigarette smoke exposure on the expression of HDAC-2, mSin3a and Mi-2α/β in the lungs of mice. We also use PI3Kγ knock-out (PI3Kγ^-/-^) and PI3K kinase dead knock-in (PI3Kδ^D910/A910^) transgenic mice to assess the impact of PI3K signalling on these components and correlate these with the restoration of glucocorticoid function.

## Materials and methods

Cigarette smoke induced GC insensitive mouse model. Studies described herein were performed under a Project License issued by the United Kingdom Home Office and protocols were approved by the Local Ethical Review Process. Both PI3Kδ kinase dead knock-in (PI3Kδ^D910A/D910A^) or PI-3Kγ knockout (PI3Kγ^-/-^) mice have been described previously [22,23]. Wild type (BALB/c; wt) and PI3Kγ^-/- ^and PI3Kδ^D910A/D910A ^mice were exposed to either cigarette smoke (5x1R3F cigarettes/day) or room-air on 3 consecutive days as previously described [24] and dosed with either budesonide (1 mg/kg) or vehicle (saline with 2% NMP) by intranasal (i.n.) administration one hour prior to exposure. Air exposed animals were subject to the exact treatment conditions and regime as smoke exposed. The budesonide dose was selected that inhibits ovalbumin induced lung inflammation [25]. Animals were sacrificed 24 hours post last exposure and tissue processing were performed as previously described [21].

### Protein extraction and Immunoblotting

Cytosolic proteins were extracted using a hypotonic lysis buffer (10 mM Tris HCl pH6.5, 0.5 mM Na Bisulfite, 10 mM MgCl_2_, 8.6% sucrose, 0.5% NP-40 phosphatase inhibitors and protease inhibitors). Nuclear proteins were extracted using a high salt extraction buffer (15 mM Tris HCL pH 7.9, 450 mM NaCl, 10% glycerol, phosphatase inhibitors and protease inhibitors) and nuclear extract salt concentrations normalised with 2 volumes of a Tris-glycerol buffer (15 mM Tris HCL pH 7.9, 10% glycerol, phosphatase inhibitors and protease inhibitors). Protein quantification was assessed by BCA assay (Perbio, Northumberland, UK). Immunoblotting and immunoprecipitation was performed as previously described [26]. All blots were stripped and re-probed for loading controls as previously described [26].

### ELISA and HDAC Activity

Both KC ELISA (RnD Systems, Adingdon, UK) and HDAC-2 activity assays (Biomol International, Exeter, UK) were performed using commercially available kits according to the manufacturer's instructions as previously [21].

### Reagents

All reagents were purchased from Sigma-Aldrich (Sigma-Aldrich, Gillingham, UK) unless otherwise stated. Phosphatase inhibitor cocktail II (Merck Biosciences, Nottingham, UK); Protease inhibitors: Complete mini cocktail inhibitor tablets (Roche Applied Science, West Sussex, UK) HDAC-2 antibody (Santa Cruz Biotechnology, CA, USA); mSin3a antibody (Abcam, Cambridge, UK); Mi2α/β antibody (Austral Biotechnology, San Ramon, CA, USA); Lamin A/C antibody (Santa Cruz); GAPDH (Abcam).

### Statistical Analysis

Data was analysed by 1 way ANOVA to determine statistical significant variance between the groups for each endpoint assessed. Statistical significance between groups was then calculated using the non-parametric Mann-Whitney U-test. All statistical analysis was performed using GraphPad Prism software using and data is expressed as mean ± SEM, differences were considered significant if *p *< 0.05.

## Results

Cigarette smoke-mediated reduction in HDAC-2 activity but not expression is associated with relative glucocorticoid insensitive inflammation in the lungs. We have previously reported that cigarette smoke exposure reduced lung HDAC-2 activity and increased lung KC levels using the same animals and samples as used in this current study [21] (Table 1). Budesonide treatment (1 mg/kg) had no impact on the expression of KC in the lungs of the smoke-exposed or sham-exposed mice (Table 1) [21] demonstrating that the cigarette smoke-mediated inflammatory response in the lungs of the mice was relatively insensitivity to glucocorticoids. PI3Kγ abolition had no impact on HDAC-2 protein expression, activity or KC levels in the lungs of the smoke-exposed animals. However, selective abolition of PI3Kδ signalling protected against smoke-induced attenuation of HDAC-2 activity and enabled glucocorticoid mediated reduction of Lung KC levels (Table 1) [21].

**Table 1:** Lung HDAC2 activity and KC expression in smoke-exposed mice [21]

	wt			PI3Kγ^-/-^			PI3Kδ^D910/A910^		
	
	Sham	Smoke	Smoke + Budesonide	Sham	Smoke	Smoke + Budesonide	Sham	Smoke	Smoke + Budesonide
Lung HDAC-2 activity (% sham)	100	53.4 ± 6.5***	53.6 ± 8.2	100	61.1 ± 6.9***	39.9 ± 3.5##	100	104.6 ± 7.3	124.7 ± 10.5

Lung KC expression (pg/mg protein)	180.1 ± 12.7	1357.2 ± 162.8***	1346.1 ± 98.1	183.5 ± 5.7	1947.1 ± 215.3***	1777.5 ± 192.6	298.2 ± 91.6	2017.5 ± 246.6***	1030.8 ± 49.6###

This table summarises key published data from the cigarette smoke-mediated glucocorticoid insensitive model (20). This data demonstrates that reduction in budensonide-mediated repression of KC is impaired the smoke-exposed animals. Abolition of PI3Kδ, but not γ signalling, restored budesonide function. This coincided with the protection of the activity of the key co-repressor HDAC-2. **p > 0.01, ***p > 0.001 (versus sham control) ##p > 0.01, ###p > 0.001 (versus smoke-exposed without budesonide).

**Figure 1:**
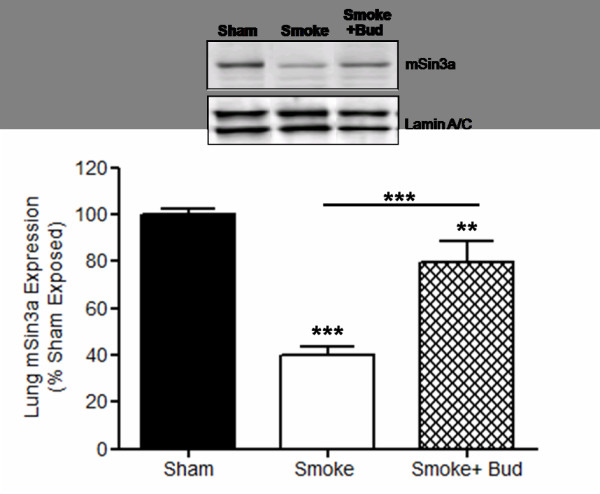
**Cigarette smoke exposure reduces lung mSin3a expression which is protected by glucocorticoid treatment**. Budesonide treatment protected the lung expression of mSin3a in smoke exposed animals. Data represents the mean ± S.E.M (n = 7-8). *** p > 0.001 compared to air exposed sham. Abbreviations; Smoke: Smoke Exposed; Bud: Budesonide.

**Figure 2:**
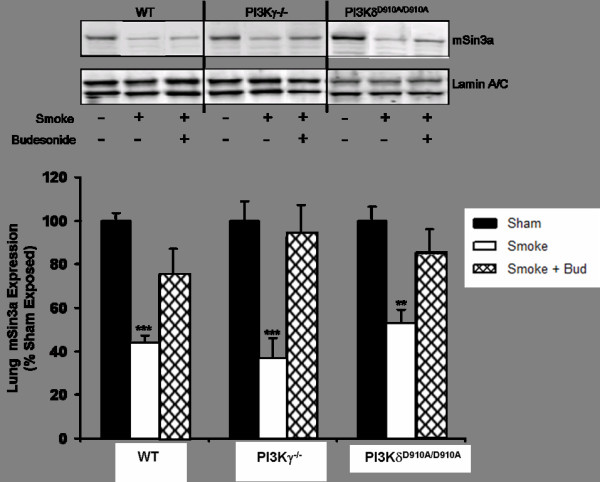
**Cigarette smoke exposure reduces lung mSin3a expression and is not protected by abolition of PI3Kγ/δ signalling in the absence of budesonide treatment**. Data represents the mean ± S.E.M (n = 7-8). *** p > 0.001 compared to air exposed sham. Abbreviations; Smoke: Smoke Exposed; Bud: Budesonide.

Cigarette smoke exposure reduces mSin3a expression. The expression of mSin3a protein was reduced by around 60% in the lungs of smoke-exposed wt animals as compared to sham-exposed animals wt (P < 0.001) (figure 1). Although budesonide treatment had no impact on HDAC-2 activity (Table 1), the expression of mSin3a protein was elevated by ~40% (P < 0.001) in the lungs of smoke-exposed wt animals treated with budesonide as compared to smoke-exposure alone (figure 1). There was no significant difference in the expression of mSin3a protein in the lungs of sham-treated PI3Kγ^-/- ^or PI3K^D910/A910 ^mice as compared to sham-treated wt mice (figure 2). There was also no difference in the reduction of mSin3a protein expression in the lung of PI3Kγ^-/- ^mice or PI3K^D910/A910 ^mice either with or without budesonide treatment as compared to smoke exposed WT (figure 2).

**Figure 3:**
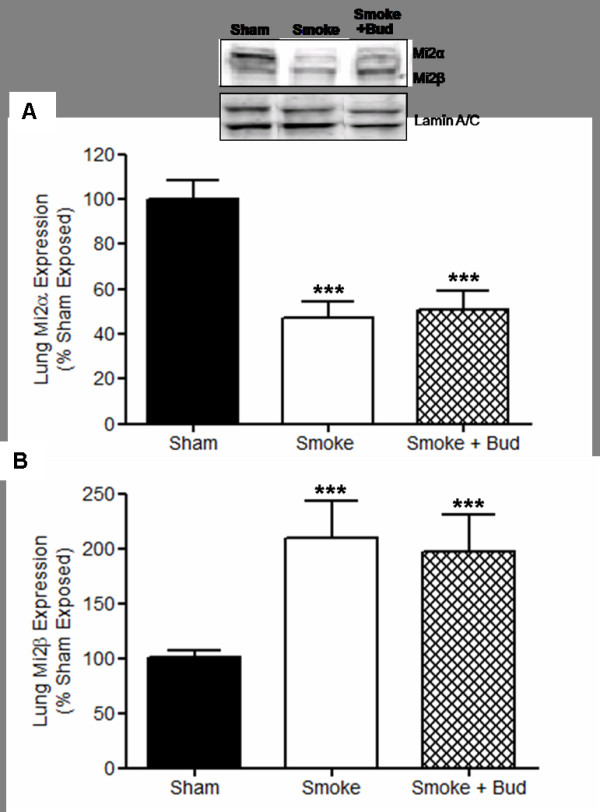
**Cigarette smoke exposure reduces Mi-2α expression but elevates Mi-2β expression in the lungs**. Cigarette smoke exposure reduced lung Mi-2α expression and this reduction was unaffected by budesonide treatment (A). Cigarette smoke exposure elevated lung Mi-2β expression and this elevation was unaffected by budesonide treatment (B). Data represents the mean ± S.E.M (n = 7-8). *** p > 0.001 compared to air exposed sham. Abbreviations; Smoke: Smoke Exposed; Bud: Budesonide.

Cigarette smoke exposure alters Mi-2α and Mi-2β expression. Consistent with the protein expression of mSin3a, the protein expression of Mi-2α was reduced by ~50% in the lungs of smoke-exposed wt animals as compared to sham-exposed wt animals (P < 0.001) (figure 3A). However, contrary to mSin3a expression, budesonide treatment had no impact on Mi2α protein expression in the lungs of smoke-exposed wt animals. In contrast, the protein expression of Mi-2β in the lungs of cigarette smoke-exposed wt animals was elevated by ~100% (P < 0.001) as compared to sham-exposed wt animals (figure 3B). Again, as for Mi-2α, the protein expression of Mi-2β in the lungs of smoke-exposed wt animals was unaffected by budesonide treatment (figure 3B).

**Figure 4:**
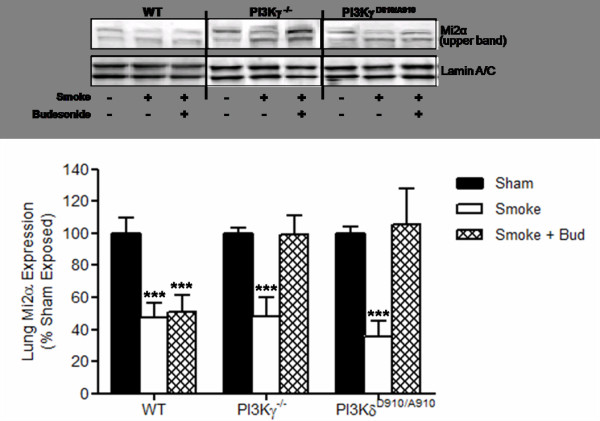
**Abolition of PI3Kγ and δ signalling enables budesonide to protect lung Mi-2α expression after cigarette smoke exposure**. Mi-2α expression levels in the lung were protected by budesonide treatment in both the PI3Kδ^D910/A910 ^mice and the PI3Kγ^-/- ^mice but not the WT mice. Data represents the mean ± S.E.M (n = 7-8). *** p > 0.001 compared to air exposed sham. Abbreviations; Smoke: Smoke Exposed; Bud: Budesonide.

There was no difference in the basal Mi-2β protein expression in the lungs of both PI3Kγ^-/- ^and PI3K^D910/A910 ^mice as compared to sham-exposed wt mice (figure 4). Furthermore, there was no difference in the elevation of Mi-2β protein expression in smoke-exposed lungs of either PI3Kγ^-/- ^or PI3K^D910/A910 ^mice with or without budesonide treatment as compared to smoke exposed wt animals with or without budesonide treatment (figure 4).

Similar to the observed data with Mi-2β, there was no alteration in the either the basal expression or cigarette smoke-mediated reduction in Mi-2α protein in the lungs of PI3Kγ^-/- ^and PI3K^D910/A910 ^mice as compared to wt sham controls (figure 4). However, budesonide treatment protected against the down regulation of Mi2α protein in the lungs of both PI3Kγ^-/- ^and PI3K^D910/A910 ^smoke-exposed mice compared to smoke exposed wt controls (Figure 4). The levels of Mi-2α protein expression in the lungs of smoke-exposed PI3Kγ^-/- ^and PI3K^D910/A910 ^mice were comparable to those seen in wt sham exposed animals (figure 4).

## Discussion

We show here for the first time that the protein expression of both Mi-2α/β and mSin3a co-repressors are altered in the lungs of mice exposed to cigarette smoke. These data are consistent with the effect of cigarette smoke on other co-repressor and repressor components in the lungs including GR and HDAC [21,26]. Both Mi-2 and mSin3a are critical components of transcriptional co-repressor complexes and changes in their expression may lead to the alteration of both the formation and targeting of these complexes. Consequently, an oxidant-mediated reduction in the protein expression of Mi-2α and mSin3a and increased protein expression of Mi2β may lead to altered gene repression. This in turn may have important implications in the development of chronic inflammation and reduction in glucocorticoid function in smoking related disease such as COPD.

Regulation of gene transcription is a highly controlled process involving the construction and recruitment of co-repressor complexes. Disruption of these complexes may lead to dysregulated gene transcription, pathophysiological changes and disease. Mi-2α/β and mSin3a coordinate the construction of co-repressor complexes to deliver transcriptional repressors including HDAC1/2 and methyl transferases to the site of gene transcription [4,5,13,14]. Both serve as co-repressor scaffold proteins that physically bridge the connections between associated co-repressors such as HDAC1/2 and the promoter bound target sequence specific transcription factor. However, relatively little is known about either the composition or the stepwise construction and targeting of the mSin3a and Mi-2 co-repressor complexes or their role in disease.

The reduction in mSin3a expression in the lungs of cigarette smoke-expose mice may reduce the capacity for the regulation of pro-inflammatory genes. In addition, cigarette smoke induces a relative reduction in glucocorticoid responsiveness in this model which is linked to a reduction in HDAC-2 activity but not expression [21]. HDAC-2 is central in the mechanisms by which GR mediates glucocorticoid induced gene repression and is proposed to be central in the development of oxidant-induced glucocorticoid insensitivity [11,12,17]. Therefore the reduction seen in mSin3a expression would likely compound the reduced functional HDAC-2 available to be recruited by GR as part of a repressor complex. This, in turn, may further impair glucocorticoid function and enhanced pro-inflammatory gene transcription. Other co-repressors such as methyltransferases, also known to be part of the mSin3a co-repressor complex are also likely to be affected. Further experimentation is needed to confirm this.

Interestingly, budesonide treatment elevated the expression if mSin3a in smoke exposed animals, although not back to the levels seen in the lungs of sham exposed controls. This may be part of a positive feedback mechanism by which glucocorticoids increase the availability of co-repressor complexes to further enhance the GR-mediated transcriptional repression.

Although Mi-2/NuRD complex contains both nucleosome remodelling and ATPase activity it also is associated with HDAC 1/2 and is involved in cell development and differentiation [4,7]. Similarly to mSin3a, the expression of Mi-2α was reduced in the lungs of smoke-exposed wt animals as compared to sham controls. This reduction, along with that seen for mSin3a, provides strong evidence that cigarette smoke exposure reduces the core components and therefore availability of co-repressor complexes for the regulation of pro-inflammatory gene transcription. To our knowledge this is the first report that oxidative stress can reduce mSin3a expression and this may play a role in the previously reported suppressive effect of oxidative stress on mSin3a-associated KLF11 activity [8].

However, in contrast to Mi-2α, the expression of Mi-2β was elevated in the smoke exposed lungs. No differences have been documented in either the cellular expression or functional roles of Mi-2α and β therefore this elevation may be a compensatory mechanism for the smoke-mediated reduction in Mi-2α expression. However, the functional impact from a shift from a Mi-2α to a Mi-2β predominant Mi-2/NuRD co-repressor is unclear. Furthermore, unlike with mSin3a, glucocorticoid treatment alone had no effect on either Mi-2α or Mi-2β protein expression in smoke-exposed lungs which indicates that mSin3a, unlike Mi-2 protein expression, is regulated by glucocorticoids in addition to its key role in transcriptional repression.

The oxidant-mediated reductions seen in HDAC-2 activity, Mi-2α and mSin3a are likely to impair the physiological regulation of pro-inflammatory gene expression and may contribute to a chronic enhanced inflammatory response seen in models of cigarette smoke exposure [21,26,27]. Cigarette smoke is the major etiological factor in the development of COPD and is also largely responsible for the elevated oxidant burden and enhanced inflammation in the lungs of COPD patients [18,28]. Therefore, an oxidant mediated reduction in these components may also play a role in the chronic enhanced inflammation in diseases seen in the lungs of COPD.

Cigarette smoke-exposure induces a relatively glucocorticoid unresponsive inflammatory response in the lungs of mice which is linked to a reduction in HDAC activity and may be a key mechanism of glucocorticoid insensitivity in COPD [11,17,21]. Both this reduction in HDAC activity and development of glucocorticoid insensitivity is abolished in transgenic mice expressing a kinase dead PI3Kδ isoform (PI3Kδ^D910/A910^) but not in PI3Kγ knock-out (PI3Kγ^-/-^) mice [21]. Here, the expression of both mSin3a, Mi-2α and Mi-2β remained unchanged in the lungs of smoke-exposed PI3Kδ^D910/A910 ^and PI3Kγ^-/- ^mice as compared to wt smoked. However, the expression of Mi-2α was protected in the lungs of the smoke-exposed PI3Kδ^D910/A910 ^and PI3Kγ^-/- ^mice treated with budesonide. Therefore, in contrast to mSin3a where budesonide treatment alone was sufficient for the protection of its expression, the budesonide-mediated maintenance of Mi-2α levels in the lungs of smoke-exposed animals appears to be dependant on the abolition of PI3Kγ/δ signalling. This suggests that cigarette smoke-mediated PI3Kγ and PI3Kδ signalling converge downstream to effect the expression of Mi-2α, perhaps through selective repression of the Chd3 gene.

Unlike HDAC activity, neither the alterations in mSin3a, Mi-2α or Mi-2β alone expression were directly consistent with the restoration of glucocorticoid responsiveness seen in this model [21]. However, the core scaffold components mSin3a and Mi-2α/β are key in bringing functional co-repressors such as HDAC1/2 to sequence specific transcription factors and thereby allowing functional repression of gene transcription and a reduction in their expression is likely to play an important role the development of reduced glucocorticoid function. Proteomic studies investigating the makeup of the GR-associated mSin3a complex under these conditions along with chromatin immunoprecipitation studies at inflammatory gene promoters are needed to confirm this.

Oxidative stress such as that derived from cigarette smoke is an extremely complex insult within the lungs and its effects, including the impairment of glucocorticoid function, are likely to be mediated though alterations in a plethora of pathways both directly and in directly. Therefore it is unlikely that a change in a single proteins activity, expression or function would be solely responsible for eliciting both a chronic and relatively glucocorticoid insensitive inflammation. However, a major hurdle in to our understanding of the roles of the various co-repressors in oxidant mediated glucocorticoid insensitivity and in disease is the lack of knowledge regarding the individual functional contributions as well as the overall function of these co-repressor complexes. Further systems biology approaches are required to develop our understanding of the roles of these co-repressor complexes and thereafter their roles in disease.

In summary cigarette smoke exposure reduced HDAC-2 activity and the expression of mSin3a and Mi-2α in the lungs of smoke exposed mice. This may contribute to the enhanced inflammatory response which is relatively insensitive to glucocorticoids. This is prevented by abolition of PI3K signalling and glucocorticoid treatment. Therefore, blockade of PI3K signalling in combination in combination with glucocorticoid treatment may provide a strategy to overcome an oxidant-induced reduction in responsiveness to the anti-inflammatory actions of glucocorticoids.

## Competing interests

The authors declare that they have no competing interests.

## Authors' contributions

JAM carried out the experimental work, participated in its design and prepared the manuscript. CSS designed and ran the in vivo model. KFC participated in the study design and preparation of the manuscript. IMA and PAK conceived of the study, participated in its design and coordination and helped in preparation of the manuscript. All authors read and approved the final manuscript.
