# Correction to: Research Cigarette Smoke Exposure Alters mSin3a and Mi-2α/β Expression; implications in the control of pro-inflammatory gene transcription and glucocorticoid function

**DOI:** 10.1186/s12950-021-00282-8

**Published:** 2021-04-23

**Authors:** John A. Marwick, Christopher S. Stevenson, Kian Fan Chung, Ian M. Adcock, Paul A. Kirkham

**Affiliations:** 1 Section of Airways Disease, National Heart & Lung Institute, Imperial College London, London, UK; 2 Respiratory Disease Area, Novartis Institute for Biomedical Research, Horsham, UK; 3 Respiratory Pharmacology, National Heart & Lung Institute, Imperial College London, London, UK

**Correction to: J Inflamm 7, 33 (2010)**

**https://doi.org/10.1186/1476-9255-7-33**

Following publication of the original article [1], an error was reported in Fig. 1 and Fig. 4.

**Fig. 1:**
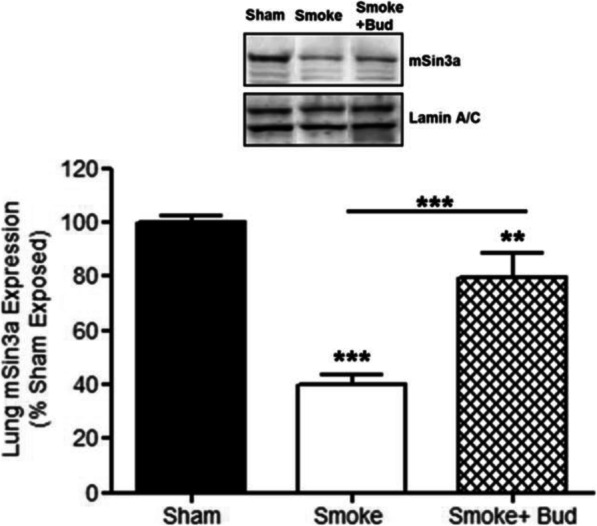
Cigarette smoke exposure reduces lung mSin3a expression which is protected by glucocorticoid treatment. Budesonide treatment protected the lung expression of mSin3a in smoke exposed animals. Data represents the mean ± S.E.M (*n* = 7–8). *** *p* > 0.001 compared to air exposed sham. Abbreviations; Smoke: Smoke Exposed; Bud: Budesonide

**Fig. 4:**
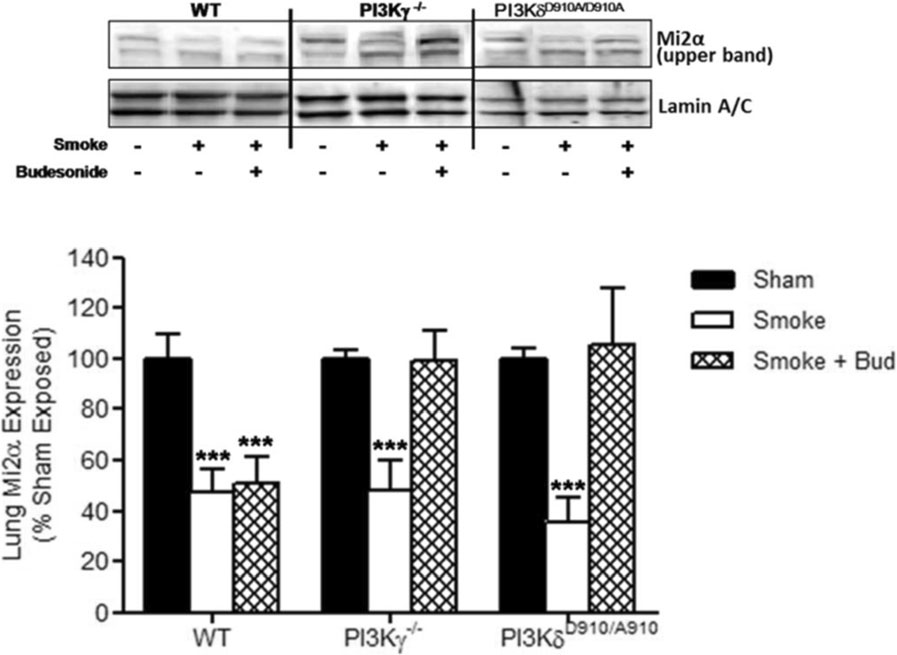
Abolition of PI3Kγ and δ signalling enables budesonide to protect lung Mi-2α expression after cigarette smoke exposure. Mi-2α expression levels in the lung were protected by budesonide treatment in both the PI3KδD910A/D910A mice and the PI3Kγ−/−mice but not the WT mice. Data represents the mean ± S.E.M (*n* = 7–8). *** *p* > 0.001 compared to air exposed sham. Abbreviations; Smoke: Smoke Exposed; Bud: Budesonide

Figure 1 correction: The incorrect immunoblot panel was included in the original submission.

The correct immunoblot panel is included in the corrected Fig. 1 below.

Figure 4 correction: The incorrect PI3KδD910A/A910A immunoblot panel was included in the original submission. The correct immunoblot panel is included in the corrected Fig. 4 below.

The authors apologize unreservedly for this oversight during the original formatting of the manuscript.
